# Association between cardiothoracic ratio, left ventricular size and systolic function in patients undergoing computed tomography coronary angiography

**DOI:** 10.3892/etm.2014.2016

**Published:** 2014-10-13

**Authors:** YINSU ZHU, HAI XU, XIAOMEI ZHU, YONGYUE WEI, GUANYU YANG, YI XU, LIJUN TANG

**Affiliations:** 1 Department of Radiology, The First Affiliated Hospital of Nanjing Medical University, Nanjing, Jiangsu 210029, P.R. China; 2 Department of Biostatistics, School of Public Health, Nanjing Medical University, Nanjing, Jiangsu 210029, P.R. China; 3 Laboratory of Image Science and Technology, School of Computer Science and Engineering, Southeast University, Nanjing, Jiangsu 210096, P.R. China

**Keywords:** dual source computed tomography, chest radiography, cardiothoracic ratio, left ventricular systolic function, left ventricular volume index

## Abstract

The present study aimed to investigate the association between cardiothoracic ratio (CTR) and left ventricular (LV) systolic function parameters in patients with or without preserved LV ejection fraction (LVEF). A total of 203 subjects suspected with coronary artery disease underwent chest radiography and dual source computed tomography coronary angiography (DSCT-CA). The LV systolic function parameters: LV end-diastolic volume index (LVEDVI), LV end-systolic volume index (LVESVI), and LVEF were measured from the DSCT-CA. The association between CTR and LV systolic function parameters was analyzed according to LVEF value (<55%, depressed LVEF group; versus ≥55%, preserved LVEF group) and CTR value (<0.5, normal range CTR group; versus ≥0.5, larger CTR group). The LVEDVI and LVESVI were higher in the depressed LVEF group compared with the preserved LVEF group (108.56±57.15 vs. 67.52±14.56 ml/m^2^, P<0.001; and 64.07±37.81 vs. 20.23±7.23 ml/m^2^, P<0.001, respectively) and lower in the normal range CTR group compared with the larger CTR group (67.10±15.00 vs. 77.30±34.32 ml/m^2^, P=0.009 and 21.94±8.96 vs. 28.97±26.54 ml/m^2^, P=0.017, respectively). Significant correlations were found between CTR and LVEDVI, and LVESVI and LVEF in the depressed LVEF group (r=0.66, P<0.001; r=0.65, P<0.001; and r=−0.46, P=0.018, respectively). However, there was no significant association detected between CTR and LV systolic function parameters in the other subgroups. The LVEDVI and LVESVI showed an inverse correlation with the LVEF in each group. Although the CTR was not a reliable indicator of LV size and systolic function in patients with preserved LVEF, it was correlated with LV size and LVEF in patients with depressed LVEF.

## Introduction

Left ventricular (LV) contractile function indicators, including ejection fraction (EF) and LV size, contribute to diagnosis (1,2), prognosis (3–5) and treatment (1,2,5,6) in patients with congestive heart failure (CHF). LVEF is possibly the optimal prognostic factor in patients with HF (2–7). The LV size is well-established as an independent predictor of cardiovascular morbidity and mortality, and is frequently used to guide patient care (8–10). Although LVEF and LV size can be readily measured using angiographic, radionuclide, computed tomography (CT), magnetic resonance imaging or echocardiographic techniques, these tests are expensive and may not be readily available in all clinical settings. Non-invasive imaging, such as chest radiography (CR), is an essential component of the diagnostic work-up and follow-up assessment of all cardiac disease.

The cardiothoracic ratio (CTR) derived from CR is traditionally used to estimate LV size (11,12) and is associated with LV systolic dysfunction (13,14). A high CTR, estimated by CR, is a marker of cardiomegaly and has been shown to be associated with poor outcomes in patients with HF and congenital heart disease (7,15–17). However, pilot studies have shown inconsistent associations between CTR and LV size (12,18–20) or systolic function (13,14,17,19). Furthermore, these study results are conflicting and have been marred by examination method bias (radiograph, radionuclide, angiographic or echocardiographic) and patient selection (mostly with depressed LVEF, congenital heart disease, CHF and other cardiac disease) bias. The association between the CTR and LV systolic function parameters in patients with preserved LVEF remains unclear.

The aim of this study was to assess the association of LV systolic function parameters evaluated by dual source CT coronary angiography (DSCT-CA) and the CTR derived from CR in patients with suspected coronary artery disease (CAD) with or without preserved LVEF. The CTR was hypothesized to have different correlations with the LV systolic function parameters according to the value of the CTR and LVEF.

## Materials and methods

### Study population

The institutional review board approved this retrospective study and waived the requirement for informed consent. A total of 203 consecutive patients were involved between June and October 2010, who were suspected of exhibiting CAD and underwent CR and DSCT-CA within three weeks. Exclusion criteria were congenital heart, pericardial, valvular heart and pulmonary hypertension diseases. The patients were divided into four subgroups according to the LVEF value (<55%, depressed LVEF group; and ≥55%, preserved LVEF group) and CTR value (<0.5, normal range CTR group; and ≥0.5, larger CTR group).

### DSCT-CA protocol

All the CT examinations were performed with a DSCT (Somatom Definition; Siemens Medical Systems, Erlangen, Germany). Four electrocardiograph (ECG) leads were attached to the chest of the patient in standard position and the ECG was continuously recorded throughout the scan. Bolus tracking was used for timing and scanning automatically started 6 sec after contrast enhancement reached 100 HU in a region of interest placed in the descending aorta. The scanner setting was as follows: Tube voltage of 120 kV and effective tube current of 380 mAs for the two tubes, and ECG pulsing window of 28–80% of the R-R interval for all the patients. The pitch was adapted for the lowest expected heart rate during scanning. Expected scan time was determined prior to scanning by scan length and pitch. Scan direction was craniocaudal starting above the coronary ostia and ending at the diaphragm below all the cardiac structures. High concentration contrast material [370 mg I/ml iopromide (Ultravist^®^); Schering AG, Berlin, Germany] was administered with a mechanical power injector (Dual Shot; MedRad Inc., Indianola, PA, USA) via a 20-gauge cannula inserted into an antecubital vein. Subsequent to contrast material delivery, 40 ml of saline solution was chased at the same rate as the contrast material. The images were reconstructed retrospectively using overlapping transversal images and a medium sharp convolution kernel (B26f) with an image matrix of 512×512 pixels, and slice thickness of 0.75 mm. A total of 10 datasets of axial images from the entire heart were reconstructed at increments of 5% of the R-R interval, starting at 5% throughout the cardiac cycle using a retrospective ECG-gated half-scan algorithm.

### Quantitative assessment of LV systolic function parameters

**Figure 1:**
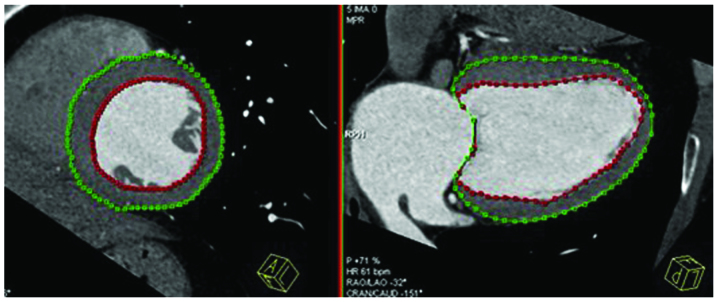
Example of the left ventricular systolic function calculated using short and long axis images from a dual source computed tomography coronary angiography.

Following reconstruction, dynamic cardiac contrast-enhanced DSCT images were transferred to an offline workstation. LV systolic function parameters were assessed: LV end-diastolic volume index (LVEDVI), LV end-systolic volume index (LVESVI), LV stroke volume index (LVSVI), LVEF and cardiac output (CO), using a semi-automatic software tool (Circulation II; Siemens Medical Systems) and calculated by the standard cube formula and indexed to body surface area. The end of the systolic and diastolic phases was determined by previewing images at the level of the mitral valve in 5% steps throughout the entire cardiac cycle (0–95% of the R-R interval). The endocardial and epicardial border contours were detected automatically and adjusted by manual tracing when necessary (Fig. 1) in the two phases. LVEF = (end diastolic − end systolic counts)/end diastolic counts × 100%. LVSV was calculated as the end diastolic minus the end systolic volume, and CO was the product of stroke volume and heart rate.

### Quantitative assessment of CTR with CR

**Figure 2:**
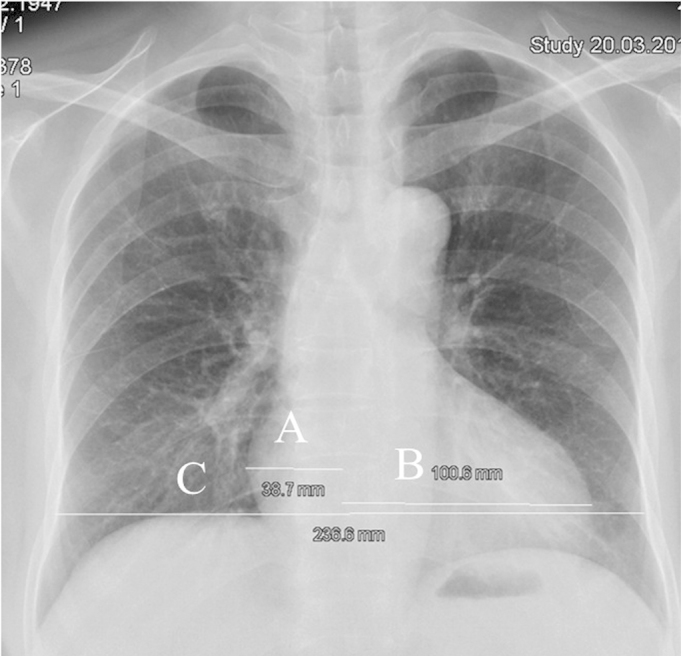
Method for determining CTR from chest radiography. The midline was defined as a vertical line drawn through the spinous processes. (A) The maximum distance from the midline to the right cardiac border was added to (B) the maximum distance from the midline to the left cardiac border. Transverse diameter of the cardiac silhouette = A+B. (C) The distance between the internal margins of the ribs at the level of the right hemidiaphragm. CTR = (A+B)/C. CTR, cardiothoracic ratio.

The CTR from the postero-anterior upright position CR was performed using standard radiological techniques. The CTR was calculated as the ratio of the maximal transverse diameter of the cardiac silhouette to the distance between the internal margins of the ribs at the level of the right hemidiaphragm (Fig. 2).

### Determination of the CTR and LV systolic function parameters

Two experienced observers blinded to the clinical data of the patient and heart rate during the scan independently measured the CTR and LV systolic function parameters of the patients. All the results were estimated by the average of two observers.

### Statistical evaluation

Continuous data were described as the mean ± standard deviation, and categorical data were described as the percentage. The analysis was stratified according to the LVEF (<55% vs. ≥55%) and CTR (<0.5 vs. ≥0.5) values. The CTR and LV systolic function parameters were compared with the Student’s t-test between subgroups. Pearson correlation and linear regression were used to describe the correlation and linear association between the CTR and LV systolic function parameters. Hypothesis test values of P<0.05 were considered statistically significant. Data management and statistical analyses were performed using SAS V9.1.3 software (SAS Institute Inc., Cary, NC, USA). The investigators had full access and responsibility for the integrity of the data.

## Results

### Patient characteristics

All the examinations were performed without technical problems, and the image quality was good for the data analysis in all cases. The study cohort consisted of 203 subjects that underwent DSCT-CA and CR, including 122 male and 81 female subjects with an age range of 29–85 (Table I).

### Comparison of the CTR and LV systolic function parameters according to the value of the LVEF and CTR

The CTR and LV systolic function parameters, such as LVEDVI, LVESVI, LVSVI, LVEF and CO, were compared according to the LVEF (<55% vs. ≥55%) and CTR (<0.5 vs. ≥0.5) values as shown in Tables II and III. No significant difference was identified for age, gender and body mass index (BMI) in all the groups with the exception of male frequency, which was higher in the normal range CTR group compared to the larger CTR group. The mean values of the LVEDVI and LVESVI of the depressed LVEF group were significantly higher than that of the preserved LVEF group (108.56±57.15 vs. 67.52±14.56 ml/m^2^, P<0.001 and 64.07±37.81 vs. 20.23±7.23 ml/m^2^, P<0.001, respectively). These two mean values in the normal range CTR group were lower compared with the larger CTR group (67.10±15.00 vs. 77.30±34.32 ml/m^2^, P=0.009 and 21.94±8.96 vs. 28.97±26.54 ml/m^2^, P=0.017, respectively). In addition, no significant difference of CTR and LVEF was identified between the groups according to the values of LVEF and CTR.

### Association between the CTR and LV systolic function parameters

The correlation between the CTR and LV systolic function parameters, including LVEDVI, LVESVI and LVEF, according to the value of LVEF and CTR are provided in Tables IV and V. Significant correlations were found between the CTR and LVEDVI, LVESVI and LVEF (r=0.66, P<0.001; r=0.65, P<0.001; and r=−0.46, P=0.018, respectively) in the depressed LVEF group. The CTR exhibited a weak correlation with LVEDVI and LVESVI (r=0.25, P<0.001; and r=0.21, P=0.002, respectively) in the overall groups. However, there was no significant association determined between CTR and LV systolic function parameters in the other subgroups.

### Association between the LV volume index and LVEF

With regard to the association between LVEDVI and LVESVI and LVEF, significant negative correlations were observed in the overall group and nearly all the subgroups with the exception of the normal range CTR group in which LVEDVI was not correlated with LVEF (Tables IV and V). Notably, the correlation was stronger between LVESVI and LVEF compared to that between LVEDVI and LVEF.

## Discussion

CR is commonly used as an initial test for the diagnosis of heart size and systolic dysfunction, particularly in general practice. The present study indicates that CTR derived from CR directly was associated with LV volume index and LVEF in patients suspected of having CAD with depressed LVEF as opposed to with preserved LVEF. To the best of our knowledge, this is the first demonstration regarding the correlation between CTR and LV systolic function parameters measured in patients according to the value of LVEF without congenital heart, pericardial, valvular heart and pulmonary hypertension diseases.

The correlation between CTR and the LV volume or diameter is known to be controversial in previous studies. Hemingway *et al* (21) and Clark *et al* (19) reported a modest to high positive correlation in patients with various heart diseases. An opposing conclusion has been reported in another study, which was that CTR was not correlated with LV volume in patients with acute chest pain (20). The present data indicated that LVEDVI and LVESVI values were higher in the larger CTR group, and showed an improved correlation between CTR and LV volume index in the depressed compared with the preserved LVEF group. One possible explanation is that the CTR measures depend on the transverse dimension of the cardiac and thoracic silhouette. The cardiac silhouette on a chest film encompasses all the contents of the pericardium. As shown in Fig. 2, the transverse dimension of the cardiac silhouette, which forms the numerator of the CTR, is predominantly affected by the right atrium size, the internal dimension of the left ventricle, the thickness of the LV wall, pericardial thickness and the contents of the pericardial space. The geometry of the thoracic shape is influenced by factors, including pneumonectasis and thoracic collapse, which are associated with pleurisy. Therefore, CTR should be regarded as an unspecific marker for LV size enlargement with questionable clinical value, particularly in congenital heart, pericardial, valvular heart and pulmonary hypertension diseases. However, patients with CAD or hypertrophic cardiomyopathy (HCM) and depressed LVEF often present with LV myocardial infarction and LV remodeling that cause dilatation of the left ventricle. When depressed patients with LVEF were evaluated and other associated diseases were excluded, the LV volume was shown to possibly be an important factor of CTR value. Of note, several studies have demonstrated a high correlation between LVEDV and LV size (20,22). Thus, the CTR can be a marker of LV size enlargement in patients with depressed LVEF rather than preserved LVEF or other associated heart diseases.

Clinicians have extrapolated that the CTR can be used to predict LV systolic function or predict the independent mortality risk in patients with CHF or congenital heart disease (2,17). The study by Cohn *et al* (13) demonstrated a modest negative correlation between CTR and EF among 584 male patients with chronic CHF and low EF enrolled in V-HeFT (vasodilator-heart failure trials) I (r=−0.27) and 758 male patients enrolled in V-HeFT II (r=−0.28). Rose and Stolberg (23) reported a negative correlation (r=−0.22) between CTR and angiographic EF among 256 subjects that underwent cardiac catheterization. The present findings have shown an improved correlation (r=−0.46) between CTR and LVEF compared to the previous studies in the patients with depressed LVEF. However, the correlation between CTR and LVEF was poor in the patients with preserved LVEF regardless of whether the CTR was small or large.

Certain clinical variables may interfere with the association between CTR and LV systolic function. Patients with HF and enlarged heart shadow, which includes cavity dimension and wall thickness, may still have preserved LVEF (24,25). For instance, an increased CTR due to hypertrophy may be associated with low, normal or high EF in patients with HF caused by hypertensive and HCM heart disease. In addition, patients with valvular heart disease may have a varied and complex association between CTR and LVEF. For example, there are distinct differences between the classic patterns of chamber enlargement and LV contractile function in patients with primary mitral stenosis and regurgitation. Another possible explanation for the weak association between CTR and EF lies with the variable distortion of the right atrial and ventricular morphological characteristics and function (26), which can occur in any given LVEF among patients with CHF. Therefore, it can be argued that LVEF can be normal in the presence of a grossly abnormal heart size. Although patients with known valvular heart disease were not included in the present study, the CTR demonstrated no significant difference according to the LVEF value and compelling evidence was obtained that CTR was not able to be reliably used to estimate LV systolic function in individual patients with preserved LVEF.

However, for patients in the later stages of CAD, dilated cardiomyopathy or HCM often present with LV myocardial infarction and remodeling, which usually cause LV dilatation and LVEF decrease. Any degree of cardiomegaly observed on the CTR is a risk factor in these patients. Although CTR is not helpful in all patients, it could play an important role in the assessment of LV systolic dysfunction and the sequential follow-up of patients with depressed LVEF.

By contrast, a strong association between LV volume index and LVEF was observed in the patients in the present study. In the majority of patients with chronic left-sided CHF, LV systolic function decreases, filling pressure increases and the chamber dilates (27). Dilation and/or hypertrophy of other cardiac chambers may also occur. Thus, patients with CHF generally have a larger LV size. A negative correlation has been described between the three-dimensional cardiac volume derived from CR and LVEF (12). Increased LV dimensions and systolic dysfunction have been shown to be powerful predictors of mortality in patients with cardiac diseases (8,28). Previously, it has been well observed that LV dilatation occurs when LV systolic function or contractile state (29) is compromised. In the present study, although the LVEDVI or LVESVI was not able to represent LV size, a negative correlation has been described between LV volume index, particularly LVESVI and LVEF.

The present study had several limitations. Firstly, this was a retrospective study and it was not possible to be certain of the standardization for CR position. A poor inspiration and the shape of the heart could make a falsely raised CTR. However, the wide scatter of the points indicated that this was not a systematic error. Secondly, in the present study, LVEDVI and LVESVI were regarded as indexes to predict LV size without considering LV wall thickness. Although there is a high correlation between LVEDVI and LV size (20,22), LV wall thickness occasionally plays an important role in LV size enlargement. Thirdly, stratification for cardiac function was not conducted in this study. Finally, the population of the depressed LVEF was smaller than that of the preserved LVEF group.

In conclusion, the present data shows an improved correlation between CTR and LV systolic function and volume index in patients with depressed LVEF compared with preserved LVEF. LV volume index is inversely correlated with LVEF in all the patients. Thus, the CTR as measured on the CR is not able to distinguish between patients with depressed or preserved LV function, but may have a role in predicting the degree of LV dysfunction and ventricular enlargement only once LV function has been reduced.

## Figures and Tables

**Table I:** Demographical and baseline clinical characteristics of the study cohort.

Characteristics	Statistical description
Age, years	60.92±11.66
Male, n	(%) 122 (60.10)
Diabetes, n (%)	56 (27.59)
Hypertension, n (%)	119 (58.62)
Smoke, n (%)	51 (25.12)
Drink, n (%)	46 (22.66)
Heart rate (bpm)	71.95±12.27
Height, cm	164.35±8.07
Weight, kg	66.91±11.12
BMI, kg/m^2^	24.68±3.21
BSA, m^2^	1.77±0,18
CTR	0.51±0.06
LVEDVI, ml/m^2^	72.78±27.89
LVESVI, ml/m^2^	25.85±20.93
LVSVI, ml/m^2^	46.87±12.60
CO, L/min	5.95±1.72
LVEF, %	66.72±11.77

Categorical variables are shown as number of patients with the percentage in parentheses. Continuous variables are shown as the mean ± standard deviation. n=203. BMI, body mass index; bpm, beats per minute; BSA, body surface area; CTR, cardiothoracic ratio of chest radiography; LVEDVI, left ventricular end-diastolic volume index; LVESVI, left ventricular end-systolic volume index; LVSVI, left ventricular stroke volume index; CO, cardiac output; LVEF left ventricular ejection fraction.

**Table II:** Comparison of the CTR and LV systolic function parameters according to the value of the LVEF for the 203 subjects.

Characteristics	LVEF <55% (n=36)	LVEF ≥55% (n=167)	P-value
Age, years	57.69±11.34	61.39±11.67	0.132
Male, n (%)	20 (76.92)	102 (57.63)	0.085
BMI, kg/m^2^	24.48±3.44	24.71±3.18	0.728
CTR	0.53±0.06	0.51±0.06	0.083
LVEDVI, ml/m^2^	108.56±57.15	67.52±14.56	<0.001
LVESVI, ml/m^2^	64.07±37.81	20.23±7.23	<0.001
LVSVI, ml/m^2^	44.07±22.89	47.28±10.32	0.226
CO, L/min	6.14±2.89	5.92±1.48	0.536
LVEF, %	42.92±8.78	70.22±7.21	<0.001

Categorical variables are shown as number of patients with the percentage in parentheses. Continuous variables are shown as the mean ± standard deviation. BMI, body mass index; CTR, cardiothoracic ratio; LV, left ventricular; LVEF, LV ejection fraction; LVEDVI, LV end-diastolic volume index; LVESVI, LV end-systolic volume index; LVSVI, LV stroke volume index; CO, cardiac output.

**Table III:** Comparison of the CTR and LV function parameters according to the value of the CTR for the 203 subjects.

Characteristics	CTR <0.5 (n=90)	CTR ≥0.5 (n=113)	P-value
Age, years	58.84±10.80	61.57±12.10	0.082
Male, n (%)	65 (72.22)	57 (50.44)	0.005
BMI, kg/m^2^	23.81±2.98	25.38±3.23	0.332
CTR	0.45±0.03	0.55±0.05	<0.001
LVEDVI, ml/m^2^	67.10±15.00	77.30±34.32	0.009
LVESVI, ml/m^2^	21.94±8.96	28.97±26.54	0.017
LVSVI, ml/m^2^	45.15±10.80	48.24±13.77	0.083
CO, L/min	5.79±1.35	6.07±1.96	0.250
LVEF, %	67.61±9.30	66.02±13.41	0.339

Categorical variables are shown as number of patients with the percentage in parentheses. Continuous variables are shown as the mean ± standard deviation. BMI, body mass index; CTR, cardiothoracic ratio; LV, left ventricular; LVEF, LV ejection fraction; LVEDVI, LV end-diastolic volume index; LVESVI, LV end-systolic volume index; LVSVI, LV stroke volume index; CO, cardiac output.

**Table IV:** Correlation between the CTR and the different measures of LV function for the 203 subjects, as stratified by LVEF.

	LVEF <55% (n=36)				LVEF ≥55% (n=167)				Overall (n=203)			
			
	CTR		LVEF (%)		CTR		LVEF (%)		CTR		LVEF (%)	
						
Group variable	r	P-value	r	P-value	r	P-value	r	P-value	r	P-value	r	P-value
LVEDVI, ml/m^2^	0.66	<0.001	−0.47	0.009	0.05	0.535	−0.15	0.023	0.25	<0.001	−0.52	<0.001
LVESVI, ml/m^2^	0.65	<0.001	−0.72	<0.001	0.04	0.566	−0.77	<0.001	0.21	0.002	−0.81	<0.001
LVEF, %	−0.46	0.018			−0.10	0.175			−0.08	0.244		

CTR, cardiothoracic ratio; LV, left ventricular; LVEF, LV ejection fraction; LVEDVI, LV end-diastolic volume index; LVESVI, LV end-systolic volume index.

**Table V:** Correlation between the CTR and the different measures of LV function for the 203 subjects who underwent CR and DSCT-CA, as stratified by CTR.

	CTR <0.5 (n=90)				CTR≥0.5 (n=113)			
		
	CTR		LVEF (%)		CTR		LVEF (%)	
				
Group variable	r	P-value	r	P-value	r	P-value	r	P-value
LVEDVI, ml/m^2^	0.20	0.053	−0.15	0.151	0.16	0.086	−0.62	<0.001
LVESVI, ml/m^2^	0.11	0.299	−0.79	<0.001	0.15	0.125	−0.85	<0.001
LVEF, %	−0.02	0.879			−0.07	0.461		

CTR, cardiothoracic ratio; LV, left ventricular; CR, chest radiography; DSCT-CA, dual source computed tomography coronary angiography; LVEF, LV ejection fraction; LVEDVI, LV end-diastolic volume index; LVESVI, LV end-systolic volume index.
